# Two-temperature LATE-PCR endpoint genotyping

**DOI:** 10.1186/1472-6750-6-44

**Published:** 2006-12-04

**Authors:** J Aquiles Sanchez, Jessica D Abramowitz, Jesse J Salk, Arthur H Reis, John E Rice, Kenneth E Pierce, Lawrence J Wangh

**Affiliations:** 1 Department of Biology, MS008, Brandeis University, Waltham, MA 02454, USA; 2 Sackler School of Medicine, Tel Aviv University, Ramat Aviv 69978, Israel; 3 Department of Human Biology, Fred Hutchinson Cancer Research Center, Seattle, WA 98019, USA

## Abstract

**Background:**

In conventional PCR, total amplicon yield becomes independent of starting template number as amplification reaches plateau and varies significantly among replicate reactions. This paper describes a strategy for reconfiguring PCR so that the signal intensity of a single fluorescent detection probe after PCR thermal cycling reflects genomic composition. The resulting method corrects for product yield variations among replicate amplification reactions, permits resolution of homozygous and heterozygous genotypes based on endpoint fluorescence signal intensities, and readily identifies imbalanced allele ratios equivalent to those arising from gene/chromosomal duplications. Furthermore, the use of only a single colored probe for genotyping enhances the multiplex detection capacity of the assay.

**Results:**

Two-Temperature LATE-PCR endpoint genotyping combines Linear-After-The-Exponential (LATE)-PCR (an advanced form of asymmetric PCR that efficiently generates single-stranded DNA) and mismatch-tolerant probes capable of detecting allele-specific targets at high temperature and total single-stranded amplicons at a lower temperature in the same reaction. The method is demonstrated here for genotyping single-nucleotide alleles of the human HEXA gene responsible for Tay-Sachs disease and for genotyping SNP alleles near the human p53 tumor suppressor gene. In each case, the final probe signals were normalized against total single-stranded DNA generated in the same reaction. Normalization reduces the coefficient of variation among replicates from 17.22% to as little as 2.78% and permits endpoint genotyping with >99.7% accuracy. These assays are robust because they are consistent over a wide range of input DNA concentrations and give the same results regardless of how many cycles of linear amplification have elapsed. The method is also sufficiently powerful to distinguish between samples with a 1:1 ratio of two alleles from samples comprised of 2:1 and 1:2 ratios of the same alleles.

**Conclusion:**

SNP genotyping via Two-Temperature LATE-PCR takes place in a homogeneous closed-tube format and uses a single hybridization probe per SNP site. These assays are convenient, rely on endpoint analysis, improve the options for construction of multiplex assays, and are suitable for SNP genotyping, mutation scanning, and detection of DNA duplication or deletions.

## Background

Amplification of double-stranded DNA occurs exponentially during the early stages of symmetric PCR, but eventually slows down and plateaus due to negative feedback between the double-stranded products and the Taq polymerase [1]. The plateau value of symmetric PCR is unsuitable for endpoint analysis of starting target numbers because subtle differences in reaction components, thermal cycling conditions, and early mispriming events cause individual replicate samples to exit exponential amplification at slightly different times. As a result, amplicon yield at the end of a symmetric PCR amplification varies significantly among replicates and the amount of accumulated amplicon at plateau does not reflect the amount of DNA present in the initial sample [2,3]. The coefficient of variation in amplicon yield among replicates can be as much as 45.1% [4].

To overcome these limitations of endpoint analysis, real-time PCR uses a variety of detection chemistries (hybridization probes or double-stranded DNA dyes) to measure the amount of each amplicon accumulating during the exponential phase of the reaction [5,6]. The thermal cycle in which a fluorescent signal is first observed above background is called the threshold cycle, or C_t _value, and is inversely proportional to the initial amount of starting templates in the reaction. Initial target numbers present in unknown samples are either measured relative to the C_t _values of known target amount standards analyzed in parallel under equivalent conditions or are measured relative to themselves in sets of replicates [5,6].

While symmetric PCR assays are quantitatively valid for measuring initial sample copy numbers over seven to eight log orders of magnitude [5,6], the reliability of individual assays makes it difficult to quantify copy number differences on the order of one to three [6,7]. However, such small changes in copy number are important because they are characteristic of aneuploidies seen *in utero*, of cancer progression (where the loss of one copy of a gene can lead to formation of a tumor), and of many other pathological conditions [7]. Many groups have attempted to remedy this limitation of real-time symmetric PCR by including internal or external reference targets of known copy number (an approach known as differential PCR and competitive PCR, respectively), followed by normalization of the end product amounts using the ratio of test and reference signals [7-11]. These strategies require extensive reaction optimization to guarantee that test and control targets amplify with similar efficiencies. In the case of competitive PCR, it is also necessary that the target and the reference control samples are similar in abundance. An alternative approach is to assay repeatedly the same sample enough times to establish a statistically reliable mean copy number for the starting material [12]. This approach is costly and assumes an abundance of sample.

In the present report, Linear-After-The-Exponential (LATE) PCR is used to generate single-stranded amplicons [13,14], and mismatch-tolerant probes are used to measure the levels of allelic variants among these amplicons via hybridization at an upper and a lower temperature at the end of PCR amplification. The resulting Two-Temperature LATE-PCR method significantly reduces the quantitative endpoint uncertainties inherent to symmetric PCR and does not require an external reference sequence for normalization. An oligonucleotide probe labelled with a single fluorophore can thereby readily distinguish between homozygous and heterozygous diploid genomes (with two copies and one copy of a particular allele, respectively) based on the fluorescent signal intensity at the end of the assay. The assay also distinguishes heterozygous samples from samples where one of the assayed alleles has undergone a duplication or loss.

This paper demonstrates the utility of the Two-Temperature LATE-PCR endpoint assay by analyzing genomes that are either homozygous or heterozygous for the normal and mutant version of the G269 allele of the human hexosaminidase A (HEXA), a single G-to-A point-mutation responsible for Tay-Sachs disease (TSD). Tay-Sachs disease is a devastating neurodegenerative disorder that occurs early in childhood due to accumulation of GM2 gangliosides in neurons as a result of HEXA protein deficiency [15,16]. We also demonstrate the general use of Two-Temperature LATE-PCR endpoint assays by genotyping two single nucleotide polymorphisms (SNP) near the human p53 tumor suppressor gene. Finally, we illustrate the ability of Two-Temperature LATE-PCR endpoint genotyping to identify allele ratios similar to those that arise from chromosomal duplications, discuss the improved multiplex detection capacity of the assay, and consider several applications of these technologies for mutation scanning and detection of chromosome trisomy or DNA deletions.

## Results

### The limitations of symmetric PCR

**Figure 1:**
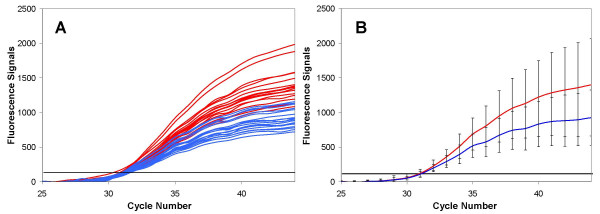
**Fluorescent signal scatter in symmetric PCR assays**. Replicate sets of homozygous normal (red lines) and heterozygous (blue lines) DNA for the TSD Δ1278 allele of the human HEXA gene were amplified using symmetric PCR. Reactions were monitored using a molecular beacon probe against the normal HEXA allele. The molecular beacon probe is allele-discriminating and recognizes 100% of the alleles in the homozygous normal samples but only 50% of the alleles in heterozygous samples. As a result, the probe fluorescence signals should theoretically be twice as intense in homozygous normal samples compared to heterozygous samples. Each reaction contained 1000 genome-equivalents of genomic DNA. **Panel A: Kinetic plots of accumulated amplification products detected by the molecular beacon probe in replicate samples**. Signal scattering among replicate samples due to stochastic factors influencing PCR in individual tubes prevents unambiguous identification of homozygous and heterozygous samples based on final fluorescence signal intensity. **Panel B: Statistical analysis of data in Panel A. **Solid lines represent the average fluorescence intensity values (red: homozygous normal samples; blue: heterozygous samples), error bars corresponds to three-standard deviations of the mean of the fluorescence signals at each amplification cycle which defines the range of fluorescence signals encompassing 99.7% of samples for each genotype. The extensive overlap between the error bars of homozygous normal and heterozygous samples demonstrates that these samples cannot be identified based solely on final fluorescence signal intensity at any amplification cycle.

Theoretically, homozygous samples with two identical alleles amplified by real-time symmetric PCR should consistently generate allele-specific probe signals that are one cycle earlier and twice as bright as probe signals from heterozygous samples with only one copy of the same allele, provided that both genotypes are used in equivalent amounts at the start of the reaction. In practice, this is not the case due to the inherent variability in amplicon yield at the plateau phase typical of symmetric PCR [2]. Figure 1A illustrates this problem by comparing the amplification plots of replicate sets of genomic DNA samples homozygous for the human wild-type HEXA gene (carrying two normal HEXA allele targets) against replicate sets of genomic DNA samples heterozygous for the TSD Δ1278 HEXA allele (carrying one normal HEXA allele target) using symmetric PCR. A molecular beacon against the normal HEXA allele was used to monitor both sets of reactions. This hybridization probe is very specific for the normal HEXA allele because the TSD Δ1278 HEXA allele is comprised of a four-nucleotide insertion [16]. Figure 1B demonstrates that the means values for the plateau levels of fluorescent signal intensities observed in the sets of homozygous and heterozygous samples in Figure 1A are, in fact, statistically different (homozygous mean relative fluorescence units (RFU) = 1397 +/- 240; vs. heterozygous mean RFU = 923 +/- 133; p < 0.0001 for n = 18 samples). However, the high coefficient of variation (CV) for the replicate reactions in each set (homozygous CV = 17.22%; heterozygous CV = 14.45%) makes it impossible to reliably genotype individual samples in each set by means of probe fluorescent intensity at plateau. Reliable genotyping via symmetric PCR therefore requires the use of two differently-colored sequence-specific probes, one for each allele [17,18]. Our goal was to develop a reliable endpoint genotyping method where the fluorescent intensity of a single-hybridization probe would reflect genome composition.

### The limitation of LATE-PCR assays

LATE-PCR is a form of asymmetric PCR that uses a limiting primer and an excess primer that differ 10–50 fold in their relative concentrations. LATE-PCR further stipulates that the concentration-adjusted melting temperature of the limiting primer, T_m_^L ^and the excess primer, T_m_^X^, must abide by the rule T_m_^L ^- T_m_^X ^≥ 0°C [13]. These primer design criteria make the exponential phase of LATE-PCR as efficient as that of conventional symmetric PCR. But, LATE-PCR assays enter linear amplification soon after they reach their C_t _values rather than continuing exponentially to the plateau phase along a stochastically determined path. Thus, while small differences exist among replicate LATE-PCR assays shortly after C_t_, these differences are amplified linearly rather than exponentially as the reaction progresses. In addition, probe-target hybridization in LATE-PCR takes place at temperatures well below the extension temperature of the reaction, thereby significantly reducing background fluorescence due to primer-dependent probe degradation early during LATE-PCR amplification [13,14].

**Figure 2:**
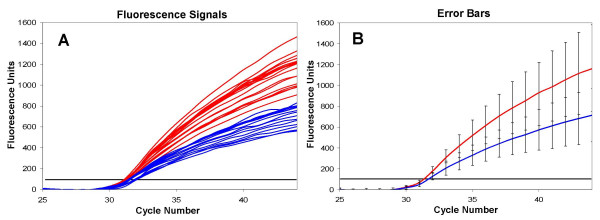
**Fluorescent signal scatter in LATE-PCR assays**. **Panel A: Kinetic plots of accumulated amplification products detected by the molecular beacon probe in replicate samples**. Replicate sets of homozygous normal (red lines) and heterozygous (blue lines) samples for the TSD Δ1278 allele (n = 18 each) were amplified using LATE-PCR and monitored in the course of the reaction using a molecular beacon probe against the normal allele. Each reaction contained 1000 genomes equivalent of genomic DNA. **Panel B: Statistical analysis of data in Panel A**. Solid lines correspond to the average fluorescence values of each replicate set (red: homozygous normal samples; blue: heterozygous samples), error bars correspond to three-standard deviations of the mean which encompasses 99.7.% of all possible samples in each replicate set distribution. LATE-PCR does reduce the overlap between the error bars of each replicate set compared to Figure 1 but still does not permit unambiguous identification of each genotype.

Figure 2 illustrates LATE-PCR amplification of replicate sets of homozygous and heterozygous HEXA samples using LATE-PCR primers derived from those used for the symmetric PCR shown in Figure 1 (see Materials and Methods). The same molecular beacon against the normal allele of HEXA used in Figure 1 was used in this experiment as well. Figure 2A shows that homozygous and heterozygous samples amplified with LATE-PCR display the expected linear kinetics and that the slopes of homozygous amplification plots are, on average, steeper than the slopes of heterozygous amplification plots. But, the spread of the final fluorescence signals for replicates of each genotype (homozygous mean RFU = 1161 +/- 145; heterozygous mean RFU = 714 +/- 92; homozygous CV = 12.51%, heterozygous CV = 12.91%) still precludes genotyping of individual samples with a 99.7% statistical certainty. This is illustrated in Figure 2B by the overlap in the range of three standard deviations for each population.

### A strategy for reducing probe signal scatter in LATE-PCR assays

One strategy for reducing probe signal scatter among replicate samples involves normalizing the allele-specific fluorescence probe signals from any given sample by the actual amount of total amplification products in that particular sample. LATE-PCR used in combination with mismatched-tolerant probes is ideally suited for this approach because these technologies permit determination of both probe-specific and total amplification products in individual reactions.

In LATE-PCR, single-stranded DNA products remain available for detection with hybridization probes at any temperature after the extension step of the thermal cycle. As a result, LATE-PCR single-stranded products can be monitored at a detection step introduced after primer extension using a mismatch-tolerant probe whose melting temperature is lower than the melting temperature of the excess primer, T_m_^X^. Because such low-T_m _probes hybridize well below the extension temperature, they can be used at concentrations that are high enough to saturate all single-stranded products without risk of interfering with primer extension and reaction efficiency [13].

Mismatch-tolerant probes hybridize to their perfectly complementary sequence at their melting temperature, but hybridize to more and more mismatched variants of those sequences as the temperature is decreased. If the temperature is lowered far enough, each probe eventually binds to the totality of variant target sequences in the reaction. Thus, for reactions that amplify specific products using single pairs of primers, the signal from a single mismatch-tolerant probe can be read at an upper temperature to measure the level of the most perfectly matched sequence and at a lower temperature to measure all related sequences present in the reaction. The ratio of probe signals collected at these two temperatures for any individual reaction normalizes the amount of single-stranded DNA products among replicate LATE-PCR assays and reveals the genotype of the sample. Hence, a homozygous normal sample probed for the normal allele generates a full strength signal at both the upper temperature and the lower temperature, while a heterozygous sample probed for the normal allele generates a half strength signal at the upper temperature and a full strength signal at the lower temperature, assuming absolute allele discrimination by the probe at the high temperature and no effect of detection temperature on probe fluorescence. We call this strategy for signal normalization Two-Temperature LATE-PCR genotyping and demonstrate its use for endpoint analysis.

In the present report, ResonSense^® ^probes [19] were used as mismatch-tolerant probes. A ResonSense^® ^probe consists of a linear fluorophore-labeled oligonucleotide used in combination with a double-stranded DNA dye. Once the probe-target hybrid forms, the resulting double-stranded DNA binds the DNA dye that then excites the probe fluorophore via fluorescence energy transfer, FRET [19]. In the experiments described here, the chosen double-stranded DNA dye (SYBR Gold I) was excited by excitation light source of the ABI 7700 at 480 nm when bound to the probe-target hybrid and served, in turn to excite the Cy5 fluorophore on the probe. As a result the probe-target hybrid emits light at 635–665 nm. Unhybridized probes remain dark because the Cy5 fluorophore is not excited at 480 nm by itself.

### Two-Temperature endpoint genotyping using LATE-PCR and ResonSense^® ^probes

**Figure 3:**
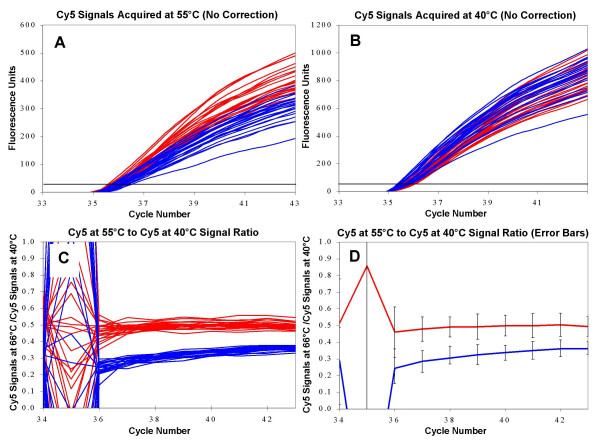
**Two-Temperature LATE-PCR Genotyping**. **Panel A: Kinetic plots of ResonSense^® ^Cy5 signals collected at 55°C, a temperature at which the probe is allele-discriminating and binds exclusively to the normal allele**. Under allele discriminating conditions, replicate homozygous normal samples (red lines) have, on the average, higher fluorescence signals than heterozygous replicates (blue lines) but signal scatter prevents identification of individual genotypes based on this type of kinetic analysis. **Panel B: Kinetic plots of ResonSense^® ^Cy5 signals collected at 40°C, a temperature at which the probe becomes mismatch-tolerant and binds both the TSD normal and G269 alleles. **Under mismatch-tolerant conditions, homozygous replicates (red lines) and heterozygous replicates (blue lines) exhibit, on the average, very similar fluorescence signals which reflect the total amount of amplification product in each tube. **Panel C: Plot of ratio of Cy5 fluorescence signals collected under allele-discriminating (Panel A) and mismatch-tolerant (panel B) conditions. **Normalization using the ratio of fluorescent signals collected at temperatures permissive for either allele-specific or mismatch-tolerant probe binding generates characteristic fluorescent signatures that are unique to each genotype. Once established, these signatures do not vary significantly as a function of the extent of the amplification reaction making them suitable for endpoint analysis (red lines, homozygous samples; blue lines, heterozygous samples). **Panel D: Statistical analysis of data in Panel C. **Red and blue lines correspond to the average fluorescence values of each replicate set of homozygous normal and heterozygous samples, respectively. Error bars correspond to three-standard deviations of the mean which encompasses 99.7% of all possible samples in each replicate set distribution. The absence of overlap between the error bars two cycles after the fluorescent signals ratio is first above background demonstrates that homozygous normal and heterozygous samples can be unambiguously identified by this method with 99.7% certainty.

Two-Temperature LATE-PCR genotyping was implemented for the TSD G269 allele of the HEXA gene by first determining the high temperature at which the ResonSense^® ^probe was allele-discriminating and then determining the lower temperature at which the probe was completely mismatch-tolerant (see Methods section). Preliminary experiments revealed these temperatures to be 55°C for the normal allele and 40°C for both alleles, respectively (data not shown). Amplification was then repeated and the fluorescent signals were measured at both 55°C and 40°C after each extension step. Figures 3A shows that under allele discriminating conditions (55°C), the means of the fluorescence signal distribution for the two sets of non-normalized plots for the homozygous and heterozygous samples are once again clearly different but the scatter among the replicates still resulted in overlapping sets of plots. In contrast, at the mismatch-tolerant temperature of 40°C the same replicate samples generated two sets of virtually identical linear plots (Figure 3B). Figure 3C demonstrates that the ratio of fluorescent signals collected at 55°C and 40°C for each sample are less scattered and clearly resolves the homozygous samples and the heterozygous samples into statistically significant different sets of assays. The mean ratio of fluorescence signals for the homozygous samples was 0.49 +/- 0.02 with a coefficient of variation of 4.08%, while the mean ratio of fluorescence signal for the heterozygous samples was 0.36 +/- 0.01 with a coefficient of variation of 2.78%. Moreover, the identity of each sample as either homozygous or heterozygous was assured with an accuracy of 99.7% (p < 0.003, Figure 3D). The results in Figure 3 also demonstrate that, as anticipated, the characteristic fluorescence ratio of the two sets of samples is constant and independent of cycle number. As a result, Two-Temperature LATE-PCR genotyping can be used as an endpoint method at virtually any thermal cycle after the end of the exponential phase of the reaction is reached. Notice, however, that the average two-temperature fluorescence ratio for the homozygous samples (0.495) is less than one and that the average two-temperature fluorescence ratios for homozygous and heterozygous samples (0.495/0.34) exhibit less than the expected two-fold difference. These deviations from the expected values are most likely due to the quenching effect of temperature on fluorescence and do not impact on the 99.7% accuracy of the assay (see Discussion).

### Fluorescence ratios generated by Two-Temperature LATE-PCR genotyping are independent of starting genome copy number

**Figure 4:**
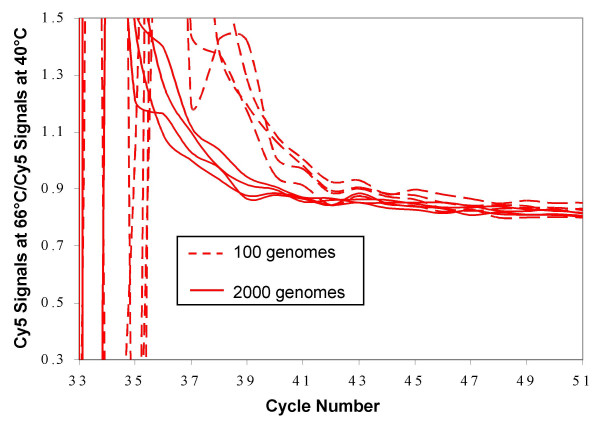
**Fluorescence ratios generated by Two-Temperature LATE-PCR genotyping are independent of starting genome copy number**. This figure shows a plot of the ratio of Cy5 readings at 50°C and 25°C derived from a LATE-PCR amplification of the genomic DNA segment containing the rs858521 [C/G] SNP monitored with a ResonSense^® ^probe against the rs858521 C allele. Red lines, homozygous CC replicates containing 2000 starting genomes; red dotted lines, homozygous CC replicates containing 100 starting genomes. Both sets of replicate homozygous samples generate the same two-temperature fluorescence ratio even though these samples differ in their amount of starting material.

Fluorescence ratios generated by Two-Temperature LATE-PCR genotyping reflect solely the intrinsic thermodynamic properties governing the binding of the fluorescent probe to its matched and mismatched targets at two different temperatures as well as the quenching effect of temperature on fluorescence intensity. As a result, fluorescence ratios indicative of genotype should be independent of the number of starting genome copies in the amplification reaction. To test this hypothesis and to showcase the applicability of Two-Temperature LATE-PCR genotyping for different amplicons, Figure 4 compares the two-temperature fluorescence ratios obtained in the course of LATE-PCR amplification for two replicate sets of samples homozygous for the C allele of the rs858521, a SNP site located in the vicinity of the human tumor suppressor gene p53. These samples consisted of either 2000 genome equivalents or 100 genome equivalents of genomic DNA at the start of the reaction. Figure 4 shows that these two sets of samples begin amplification and reach the same fluorescence ratio shortly after each set of samples reached its own C_t _value. A single endpoint reading taken at any point after cycle 43 in Figure 4 identifies these samples as having identical genotypes (i.e., identical two-temperature fluorescence ratios) despite their differences in starting copy number. We conclude that Two-Temperature LATE-PCR genotyping is robust because it allows for endpoint analysis independently of the starting number of genomes present in the sample.

### Detection of imbalanced allele ratios using Two-Temperature LATE-PCR

**Figure 5:**
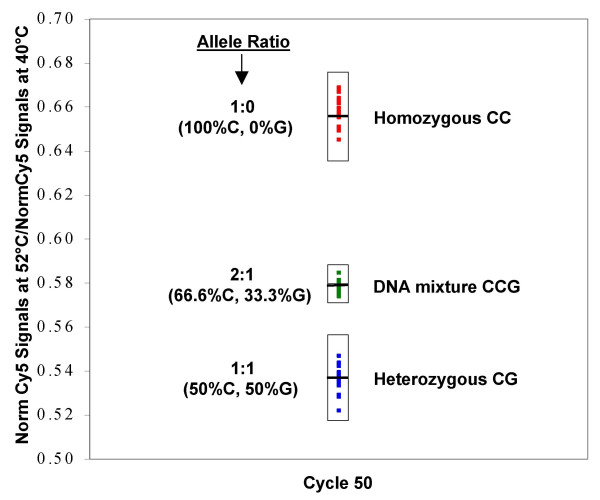
**Identification of imbalance allele ratios using the Two-Temperature LATE-PCR endpoint assay**. Replicate samples (n = 16) containing 1000 genome-equivalents of heterozygous TSD G269 DNA (1:1 C to G allele ratio) or a DNA mixture containing a 2:1 ratio of the same two alleles (see Materials and Methods) were analyzed by Two-Temperature LATE-PCR using primer/probe pairs for the TSD G269 polymorphism. Fluorescence signals were collected prior to LATE-PCR amplification at 70°C and after LATE-PCR amplification at 52°C and 40°C to calculate the normalized endpoint fluorescence ratios (see Materials and Methods). Blue dots, heterozygous (1:1 C-toG allele ratio) fluorescence ratios; green dots, DNA mixture (2:1 C-to-G allele ratio). The black boxes represent the boundary of three-standard deviations on either side of the mean of replicate control samples signals for each genotype and statistically define the 99.7% confidence interval for identification of each genotype.

We evaluated the ability of Two-Temperature LATE-PCR to distinguish between TSD G269 heterozygous samples exhibiting a balanced allele ratio of 1:1 and samples exhibiting an imbalanced allele ratio of 2:1 for the same alleles. The latter were prepared by mixing TSD G269 homozygous and TSD G269 heterozygous DNA in the proper amounts (see Materials and Methods). Such DNA mixture serves as a model for cases of chromosomal DNA duplication involving one of the alleles of a heterozygous marker. Figure 5 demonstrates that Two-Temperature LATE-PCR can readily distinguish replicate samples with balanced allele ratios (1:1) from replicate samples with an imbalanced allele ratio of 2:1 with 99.7% accuracy provided that the probe signals are further normalized for their background fluorescence values at the start of the assay (see Materials and Methods). The same results were obtained with samples consisting of 1:2 allele ratios (not shown). Preliminary experiments revealed that the assay could resolve allele-imbalances as small as 1.5:1 at the present (Sanchez, unpublished observations). These results suggest that Two-Temperature LATE-PCR assays may be capable of identifying allele imbalances that occur due to chromosome trisomies and gene duplications (see Discussion).

### Application of Two-Temperature LATE-PCR for endpoint genotyping

**Figure 6:**
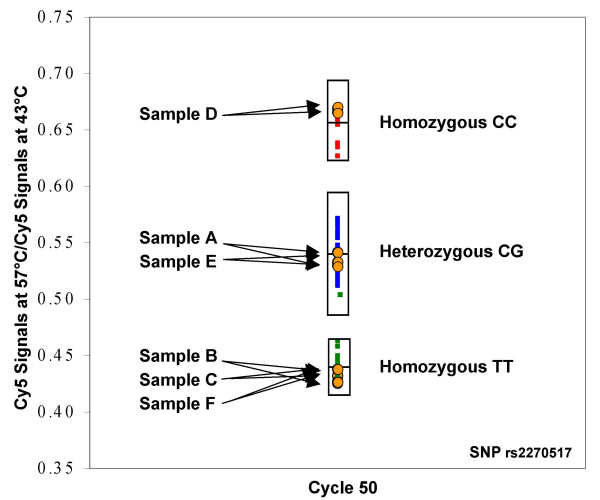
**Two-Temperature LATE-PCR endpoint genotyping of the rs2270517 SNP in blinded DNA samples**. Control replicate DNA samples corresponding to various known genotypes for the rs2270517 SNP were analyzed by Two-Temperature LATE-PCR using primer/probe pairs for the rs2270517 (C/T alleles) and a mismatch-tolerant probe specific for the C allele at high temperatures. Fluorescence signals were collected after LATE-PCR amplification at 57°C and 45°C and were used to calculate fluorescence ratios. In an initial experiment, replicate samples of each rs2270517 genotype (n = 26) were used to determine the distribution of fluorescence ratios corresponding to each genotype. Red dots indicate homozygous CC control samples, blue dots indicate the heterozygous CT control samples, and green dots indicate homozygous TT control samples. The boxes represent the boundary of three-tandard deviations on either side of the mean of replicate control samples signals for each genotype. Therefore, each box defines the range of fluorescence ratios statistically encompassing 99.7% of all possible samples of any given genotype in this assay. In two subsequent separate experiments, samples of known rs2270517 genotype that were blinded to the experimenter (coded samples A, B, C, D, E, and F in the figure) along with a set of control samples of known genotypes were processed to determine their fluorescence ratios in duplicate. These fluorescence ratios are shown in the figure as orange circles labelled with the sample designation; some of the circles from individual samples overlap in the figure. The data shows that the fluorescence ratios from each of the blinded samples fell within one of the defined ranges of normalized control fluorescence ratios. The same was observed for the set of control samples (shown as red, blue, or green dots according to genotype as specified above). There was 100% concordance in the genotype assignment when the blinded samples were decoded.

To illustrate the use of the Two-Temperature LATE-PCR for endpoint genotyping, blinded samples of known genotypes for an interrogated SNP site were tested with this assay. The particular SNP site genotyped in those experiments, rs2270517, consists of a C/T polymorphism located in the vicinity of the human p53 gene. In each instance, samples were amplified with LATE-PCR primers in the presence of a ResonSense^® ^probe against the rs2270517 C polymorphism. At the end of the assay, fluorescent readings from the hybridization probe were collected at the two appropriate temperatures and then used to calculate fluorescence ratios (see Materials and Methods for details). Replicate control samples (n = 26) of known genotypes for the rs2270517 SNP (homozygous CC, heterozygous CT, and homozygous TT) were initially processed to determine the distribution of normalized fluorescence ratios corresponding to each genotype. This data was then used to define the 99.7% confidence intervals for the distribution of fluorescence ratios corresponding to each genotype based on three standard distributions around the mean fluorescence ratio for each genotype (see boxes in Figure 6). Then, in two separate experiments, sets of control samples and purified DNA samples (100 genome-equivalents) from six different individuals whose rs2270517 genotypes were previously established by conventional methods but blinded to the experimenter were analyzed to generate duplicate endpoint fluorescence ratios for each sample. Figure 6 shows that the fluorescence ratios from each of the blinded samples fell unambiguously within one of the defined ranges of normalized control fluorescence ratios. The same was observed for the set of control samples (not shown). There was 100% concordance in the genotype assignment when the blinded samples were decoded.

## Discussion

LATE-PCR efficiently generates single-stranded products that remain available at the end of the reaction for hybridization over a broad temperature range [13]. Each Two-Temperature LATE-PCR endpoint assay uses a single mismatch-tolerant probe to detect both allele-specific sequences at an upper temperature and the totality of all sequence variants at a lower temperature. Two-Temperature LATE-PCR endpoint assays thus reduce the quantitative uncertainties inherent to symmetric PCR endpoint assays [2] by constructing ratios of fluorescent signals acquired at these two temperatures that are characteristic of the targets involved, regardless of their absolute concentrations. Two-Temperature LATE-PCR endpoint assays are particularly robust and powerful because fluorescence ratios indicative of genotype are independent of the starting amount of DNA present in the sample or the extent of product accumulation past the C_t _value. As a result, there is no need to adjust the starting amounts of materials used in the assay or to use a fixed endpoint for analysis. The reason for the high flexibility of Two-Temperature LATE-PCR assays is that the critical temperatures required for the probe to be either allele-discriminating or mismatch-tolerant are defined solely by the particular nucleotide composition of the probe under the condition of probe excess over target.

Genotyping using the Two-Temperature LATE-PCR method is possible because the two-temperature fluorescence ratios reflect the fraction of amplification products that carry the interrogated allele in any given genotype. In theory, samples homozygous for an allele interrogated by the probe should yield a two-temperature fluorescence ratio of one because the probe detects the totality of amplification products at both high (allele-discriminating) and low (mismatch-tolerant) temperatures. According to the same logic, samples heterozygous for the interrogated allele should yield a fluorescent signal ratio of 0.5 because the probe detects half of the amplification products at a high (allele-discriminating) temperature and the totality of the products at a low (mismatched-tolerant) temperature. In practice, the two-temperature fluorescence ratio of homozygous samples is less than one and the two-temperature fluorescence ratios of homozygous and heterozygous samples exhibit less than the expected two-fold difference probably because of the quenching effect of temperature on fluorescence intensity (a phenomenon discussed in [20]). Correction for the quenching effect of temperature on fluorescence intensity is difficult in the case of ResonSense^® ^probes because probe signal intensity is determined by both the fluorophore and the SYBR Gold I DNA dye [19]. We are currently designing mismatch- tolerant probe that rely on a single fluorophore to overcome this problem. However, even the current semi-quantitative design of Two-Temperature LATE-PCR is capable of successfully distinguishing heterozygous genomes with a 1:1 ratio of two alleles from DNA mixtures comprised of 2:1 and 1:2 ratios of the same two alleles. Allelic imbalances such as these occur in cases of chromosome or gene duplication due to cancer and other diseases [7].

Two-Temperature LATE-PCR endpoint assays are currently being developed in our laboratory for identification of chromosome trisomies and gene duplication at numerous SNP sites in the genome. Two-Temperature LATE-PCR is also suitable for detection of imbalance allele ratios on the order of 1.5:1 that are generated by loss of heterozygosity (deletion) events involving tumor suppressor genes in human pre-malignant cells in the presence of a two-fold excess contaminating normal cells (Sanchez *et al.*, unpublished data).

In order to address the inter-assay variability of the Two-Temperature LATE-PCR method, we showed in three separate experiments that the fluorescence ratios of samples of known genotypes fall unambiguously within a defined range of predetermined normalized control fluorescence ratios unique to each genotype. In the first experiment, control replicates sets for each genotype were used to define the 99.7% confidence intervals for the distribution of fluorescence ratios corresponding to each genotype based on three standard distributions around the mean fluorescence ratio unique to each genotype. In two subsequent experiments, samples whose genotype was blinded to the experimenter as well as additional control samples were analyzed to determine their fluorescence ratios. In each case, fluorescence ratios from each of the blinded samples and control samples fell unambiguously within one of the defined ranges of normalized control fluorescence ratios. There was 100% concordance in the genotype assignment when the blinded samples were decoded. This experimental strategy addresses the problems associated with inter-assay variation of fluorescence ratios. Until more different examples of Two-Temperature LATE-PCR assays are evaluated for inter-assay variability, however, we recommend the use of the use of reference samples of known genotypes as internal controls for every genotyping experiment. Inter-assay variation is not a concern when such internal reference controls are present given the low intra-assay variability demonstrated here.

The difficulties associated with performing endpoint genotyping based on probe signal intensity using symmetric PCR that are successfully addressed by the two-temperature genotyping method are inherent to the amplification process itself. Stochastic differences among replicate samples exiting exponential amplification result in significant differences in the final yield of double-stranded DNA products. As a result, the final probe signal intensity does not reflect the starting number of alleles in the sample [2]. Symmetric PCR amplification can be examined in real-time to circumvent the problem of endpoint product yield scatter among replicates [5,6]. Two-Temperature LATE-PCR assays permit semi-quantitative endpoint detection of allele-ratios at endpoint and do not require real-time PCR equipment; in principle, Two-Temperature LATE-PCR could be performed using only a standard thermal cycler and a temperature-regulated fluorimeter. In addition, the use of a single mismatch-tolerant probe makes Two-Temperature LATE-PCR assays easier to design.

Two-Temperature LATE-PCR also has distinct advantages over current qualitative approaches to closed-tube endpoint SNP genotyping that employ symmetric PCR and allele-specific Taqman or molecular beacon probes of two or more different colors [17]. In those approaches, genotype is qualitatively determined at endpoint by the mixture of probe signal colors present following PCR [17,18]. The requirement of two colors to define genotype restricts the number of SNP sites that can be genotyped in multiplexed fashion in single-tube format given the limit on the number of fluorophores than can be simultaneously monitored in today's fluorimeters. In contrast, Two-Temperature LATE-PCR genotyping exhibits a greater multiplex detection capacity because each complete genotyping assay requires only a single-color hybridization probe. In addition, Two-Temperature LATE-PCR is capable of identifying the presence of allele ratio imbalances semi-quantitatively, a task that cannot be done by conventional endpoint genotyping relying on color probes (i.e., color probes yield the same mixture of colored signals whether the sample is comprised of heterozygous cells or mixtures of heterozygous and homozygous cells).

Alternative approaches for endpoint symmetric PCR genotyping are more quantitative but also more cumbersome to use. These assays (known as competitor PCR, differential PCR) rely on internal or external control standards that serve as references with which to normalize test signals [7-11]. Such normalization requires internal control standards with similar amplification efficiencies as the test target or external controls. In the case of exogenous controls, it is also important that the control and test samples have the same number of targets. Two-Temperature LATE-PCR does not require an external reference sequence and corrects for variations in total amplification product yield among replicate PCR samples by normalizing each sample to itself using mismatch-tolerant probes.

Two-Temperature LATE-PCR using a single mismatch-tolerant probe may also be suitable for mutation scanning. If a sequence variant allele arises at a particular homozygous locus the quantity of the bound probe at the end of the reaction would change thereby revealing the presence of an altered, yet unknown sequence. Such "gain of heterozygosity" can then be further analyzed by directly sequencing the single-stranded DNA of the LATE-PCR assay using either Pyrosequencing [21] or the "Dilute-N'-Go" dideoxysequencing methods developed for this purpose (Sanchez and Reis, unpublished).

## Conclusion

Two-Temperature LATE-PCR permits construction of robust and convenient endpoint genotyping assays. These assays are carried out in a single tube format, use a single hybridization probe, give consistent results for various amounts of starting templates in the reaction, and are indicative of genotype regardless of how many cycles of linear amplification have elapsed. This methodology is suitable for SNP genotyping and is currently being used to construct novel, clinical compatible applications for endpoint detection of chromosomal gene duplication and deletions for cancer and prenatal genetic diagnosis.

## Methods

### Primers, probes sequences, genomic targets, and amplification conditions

Primers, molecular beacon probes, and thermal cycle conditions for symmetric and LATE-PCR amplification of the human HEXA normal and TSD Δ1278 alleles were as described [13]. Purified genomic DNA homozygous and heterozygous for the TSD Δ1278 HEXA and the TSD G269 alleles were obtained from the Coriell Cell Repositories (Camden, NJ; nucleic acid samples NA11852 – homozygous normal for TSD G269, NA03441 – homozygous normal for TSD Δ1278 HEXA, and NA03575 – heterozygous for both TSD Δ1278 and TSD G269). Genomic DNA samples with various genotypes for the rs858521 and rs2270517 SNP site were a kind gift from Dr. Brian Reid (Divisions of Human Biology and Public Health Sciences, Fred Hutchinson Cancer Research Center, Seattle, WA). These samples are also available from the Coriell Cell Repository (nucleic acid samples NA10839 – homozygous CC-, NA10830 – heterozygous CG-, NA07348 – homozygous GG- for rs858521 SNP; nucleic acid samples NA18524 – homozygous CC-, NA18526 – heterozygous CT-, NA18562 – homozygous TT- for rs2270517 SNP).

LATE-PCR primers and ResonSense^® ^probe sequences for TSD G269 locus were:

TSD G269 limiting primer: 5' CGAGGTCATTGAATACGCACGGCTCC 3'

TSD G269 excess primer: 5' TAACAAGCAGAGTCCCTCTGGT 3'

TSD G269 G allele mismatch-tolerant probe: 5' [Cy5]GGGACCAGGTAAGAA [Phos]

Primers and ResonSense^® ^probe sequences for rs858521 SNP site (C/G alleles) located 41.5 Kbp upstream of the p53 gene in human chromosome 17 were:

rs858521 limiting primer: 5' TCCCCAGAGCCCAGCCGGTGTCATTTTC 3'

rs858521 excess primer: 5' CAATCCCTTGACCTGTTGTGGAGAGAA 3'

rs858521 C allele mismatch-tolerant probe: 5' [Cy5] CTTCAGCTCAAACAATA [Phos].

Primers and ResonSense^® ^probe sequences for rs2270517 SNP site (C/T alleles) located downstream of the p53 gene in human chromosome 17 were:

rs2270517 excess primer: 5' GAGGCAGCCCGAGCAATG 3'

rs2270517 limiting primer: 5' GGTCAGCGCCGGGCTGCAAGTGTAGA 3'

rs2270517 C allele mismatch-tolerant probe: 5' [Cy5] AGCGGGTGGTAG [Phos].

Amplification reactions for the TSD G269 and rs858521 amplicons consisted of 1× Platinum Taq Buffer (Invitrogen, Carlsbad, CA), 3 mM MgCl_2_, 250 nM dNTP, 25 nM (for TSD G269) or 50 nM (for rs858521) limiting primer, 1000 nM corresponding excess primer, 0.24× Syber Gold I (Invitrogen, Carlsbad, CA), 1.25 units Platinum Taq (Invitrogen, Carlsbad, CA), 0.1× Primesafe (for TSD G269; Smiths Detection, Edgewood, MD) or 0.2× Primesafe (for rs858521; Smiths Detection, Edgewood, MD), 2.4 μM of the respective ResonSense^® ^probe, and 100–1000 genome-equivalent of human DNA in a volume of 25 μl. Amplification reactions for the rs2270517 amplicon consisted of 1× Platinum Taq Buffer (Invitrogen, Carlsbad, CA), 3 mM MgCl_2_, 250 nM dNTP, 50 nM limiting primer, 1000 nM corresponding excess primer, 0.24× SyberGold I (Invitrogen, Carlsbad, CA), 1.25 units Platinum Taq (Invitrogen, Carlsbad, CA), 0.1× Primesafe (Smiths Detection, Edgewood, MD), 2.4 μM rs2270517 ResonSense^® ^probe, and 100 genome-equivalents of human DNA in a volume of 25 μl. Amplification was carried out in an ABI Prism 7700 Sequence Detection System (Applied Biosystems, Foster City, CA). The thermal cycling profile for TSD G269 amplification was 95°C for 3 min; followed by 15 cycles of 95°C for 10 sec, 65°C for 20 sec, and 72°C for 20 sec; then 30 cycles of 95°C for 10 sec, 65°C for 20 sec., 72°C for 20 sec, 55°C for 20 sec. 40°C for 20 sec with SYBR Gold I fluorescence collection at the 72°C step and Cy5 fluorescence collection at the 55°C and 40°C steps. The thermal cycling profile for rs858521 amplification was 95°C for 3 min; followed by 18 cycles of 95°C for 10 sec, 66°C for 10 sec, and 72°C for 20 sec; then 33 cycles of 95°C for 10 sec, 65°C for 10 sec., 72°C for 20 sec, 55°C for 20 sec. 25°C for 20 sec with SYBR Gold I fluorescence collection at the 72°C step and Cy5 fluorescence collection at the 55°C and 25°C steps. The thermal cycling profile for rs2270517 amplification was 95°C for 3 min; followed by 18 cycles of 95°C for 10 sec, 66°C for 20 sec, and 72°C for 20 sec; then 32 cycles of 95°C for 10 sec, 66°C for 20 sec., 72°C for 20 sec. In the latter case, Cy5 fluorescence signals were acquired at 57°C and 45°C after LATE-PCR amplification for two-temperature endpoint genotyping. Calibration of the ABI 7700 for use of the Cy5 ResonSense^® ^probe and SYBR Gold I dye was performed according to the manufacturer's instructions using 1.25 μM of the G269 probe (for the ResonSense^® ^probe) and 100 ng human genomic DNA stained with 0.24× SYBR Gold I (for SYBR Gold I).

DNA mixtures containing a 2:1 imbalance allele ratio of C and G alleles at the TSD G269 locus were constructed by mixing homozygous normal CC and heterozygous GC TSD G269 DNA samples of matched concentrations at a 1:2 ratio. The concentration of the mixture was then adjusted to 1000 genomes-equivalents/ul (6 ng/ul)

### Mismatch-tolerant probe design criteria

It is important that the mismatch-tolerant ResonSense^® ^probe has minimal secondary structure at lower temperatures to prevent the probe from acquiring any double-stranded nature that would bind any SYBR Gold and yield Cy5 fluorescence in the absence of target binding. Typically, the probe is designed to have a melting temperature (T_m_) with the perfectly complementary target that is at least 10°C above the T_m _of the probe with the mismatched target, and is also at least 5°C below the T_m _of the limiting primer. Also, the ResonSense^® ^probes used in this study were labelled at the 5'-end with Cy5 and phosphorylated at the 3' end to prevent them from serving as a primer as described in the original publication [19]. An alternative, simpler design would be to use Cy5-labeled probes labelled at the 3' end instead.

### Determination of optimal detection temperatures for Two-Temperature LATE-PCR genotyping

Determination of the optimal upper and lower temperatures for signal acquisition to achieve maximal allele discrimination and detection of total sequence variants with mismatch-tolerant probes respectively was done empirically for each probe-target combination by collecting probe fluorescent signals at 5°C intervals, starting 9°C–10°C below the T_m _of the limiting primer down to 25°C. The optimal upper temperature shows the greatest difference between the homozygous and heterozygous genotypes of the interrogated allele, while the most preferred lower temperature shows the least difference between the homozygous forms of the interrogated and the non-interrogated allele (see Figure 3).

### Probe signal normalization

Cy5 fluorescent probe signals collected at the various temperatures were exported to Microsoft Excel from the ABI Sequence Detector software built into the ABI 7700 as clipped files. Although the data presented in this paper was exported after baseline correction according to the parameters of the ABI Sequence Detector software, clipped fluorescence values with no baseline correction can be exported as well (this was the case for the analysis shown in Figures 5 and 6 where baseline could not be determined since it is an endpoint assay). For two-temperature genotyping, we calculated for each sample the ratio of Cy5 fluorescence value collected at the upper temperature at each amplification cycle to the Cy5 fluorescent signal collected at the lower temperature for the same sample. For discrimination of heterozygous and DNA mixtures containing 2:1 allele ratios, we substrated from the fluorescence signals collected at the upper and the lower temperatures at the end of the assay for each sample the background fluorescence signals of the probe collected at 70°C prior to the start of the assay for the same sample and then calculated fluorescence ratios. Maximum discrimination of allele imbalances occurs when the fluorescence signals were collected at 52°C instead of 55°C.

### Statistical analysis

The variability in final fluorescent signals among replicate samples was measured by calculating the mean and standard deviation (S.D.). The spread of the fluorescent signals was expressed as the coefficient of variation or CV (i.e., the standard deviation expressed as a percentage of the mean). The assay was considered 99.7% accurate if the distribution of fluorescence signals from homozygous and heterozygous replicate samples were separated by more than three S.D. of the mean of each distribution. Statistical analysis of replicate symmetric PCR samples was done using the two-sample test for independent analysis with unequal variances. The probability value p refers to the null hypothesis (*i.e., *that the distribution of the means of each dataset are the same). Analysis was performed using Microsoft Excel or an online statistical calculator [22].

## Authors' contributions

JAS designed the study, demonstrated the notion of Two-Temperature LATE-PCR genotyping, and drafted the manuscript. JDA performed the G269 and rs858521 genotyping experiments. JJS conceived the rs858521 and rs2270517 genotyping experiments, contributed to statistical analysis of the data, and helped draft the manuscript. AHR provided crucial insights which led to the notion of two-temperature fluorescence ratios. JER designed, tested, and optimized the normal HEXA molecular beacon probe and the Tay-Sachs Δ1278 and G269 symmetric and LATE-PCR assays. KEP participated in the design of mismatch-tolerant probes, provided insights into their optimal use, and helped draft the manuscript. LJW participated in the design and coordination of the project and drafted the manuscript. All authors read and approved the final manuscript.
